# An international estimate of the prevalence of differing visual imagery abilities

**DOI:** 10.3389/fpsyg.2024.1454107

**Published:** 2024-10-15

**Authors:** David J. Wright, Matthew W. Scott, Sarah N. Kraeutner, Pamela Barhoun, Maurizio Bertollo, Mark J. Campbell, Baptiste M. Waltzing, Stephan F. Dahm, Maaike Esselaar, Cornelia Frank, Robert M. Hardwick, Ian Fuelscher, Ben Marshall, Nicola J. Hodges, Christian Hyde, Paul S. Holmes

**Affiliations:** ^1^Department of Psychology, Manchester Metropolitan University, Manchester, United Kingdom; ^2^School of Kinesiology, University of British Columbia, Vancouver, BC, Canada; ^3^Department of Psychology, University of British Columbia, Kelowna, BC, Canada; ^4^Cognitive Neuroscience Unit, School of Psychology, Deakin University, Geelong, VIC, Australia; ^5^BIND Center, Department of Medicine and Aging Sciences, Università degli Studi “G. d’Annunzio”, Chieti-Pescara, Italy; ^6^Department of Physical Education and Sport Sciences, University of Limerick, Limerick, Ireland; ^7^Centre for Sport Leadership, Maties Sport, Stellenbosch University, Stellenbosch, South Africa; ^8^Institute of Neuroscience, Université catholique de Louvain, Louvain-la-Neuve, Belgium; ^9^Department of Psychology, Faculty of Psychology and Sports Sciences, University of Innsbruck, Innsbruck, Austria; ^10^Department of Life Sciences, Manchester Metropolitan University, Manchester, United Kingdom; ^11^Human Movement Science Group, Department of Sports Science, University of Bremen, Bremen, Germany; ^12^Department of Sport and Exercise Sciences, Manchester Metropolitan University, Manchester, United Kingdom

**Keywords:** aphantasia, hypophantasia, hyperphantasia, VVIQ, visual imagery, imagery ability

## Abstract

The aim of this research was to establish prevalence estimates for aphantasia, hypophantasia, typical imagery ability, and hyperphantasia in a large multi-national cohort. In Study 1, the Vividness of Visual Imagery Questionnaire was completed by 3,049 participants. Results indicated prevalence estimates of 1.2% for aphantasia, 3% for hypophantasia, 89.9% for typical imagery ability, and 5.9% for hyperphantasia. In Study 2, to replicate these findings in a larger sample, the Study 1 data were combined with openly available data from previous prevalence studies to create a total sample of 9,063 participants. Re-analysis of this data confirmed prevalence estimates of 0.9% for aphantasia, 3.3% for hypophantasia, 89.7% for typical imagery ability, and 6.1% for hyperphantasia. These robust and up-to-date estimates provide enhanced clarity to researchers regarding the prevalence of differing visual imagery abilities and provide a platform for future studies exploring the role of visual imagery in various cognitive and behavioral tasks.

## Introduction

Visual mental imagery is the process of ‘seeing’ without actually perceiving an immediate sensory stimulus (Kosslyn et al., 1995). For example, if asked to imagine a horse, a sunset, or another person, most people will generate vivid and well-defined corresponding mental images and can “see” these things in their mind despite their perceptual absence. This ability plays an important role in various fundamental social, cognitive, and motor processes, including facial recognition (Dance et al., 2023), memory (Dawes et al., 2020), and perspective taking (Ward et al., 2022). Visual imagery is also a key aspect of many therapeutic psychological interventions (Saulsman et al., 2019) and is involved in many psychological experimental paradigms (e.g., Barhoun et al., 2019). Although the generation of visual imagery is a seemingly simple cognitive task for many individuals, it is well established that individual differences in visual imagery exist (Zeman et al., 2015).

The earliest evidence of differences in visual imagery ability can be traced back almost 150 years to Galton’s (1880) “Breakfast Table” study. Galton instructed 100 intellectuals to generate a mental image of the scene of their breakfast table that morning and to describe in writing the clarity of their mental image. Some reported the ability to generate images that were as clear as the actual scene, while others described dim and poorly defined mental images, or described an inability to generate any form of mental image. Self-report questionnaire measures have since been developed to quantify these differences in visual imagery ability, with the Vividness of Visual Imagery Questionnaire (VVIQ; Marks, 1973) being the most commonly used (Blomkvist and Marks, 2023). The VVIQ is a 16-item self-report scale that instructs participants to imagine a variety of scenarios, such as a shop front or country scene. Participants then rate the vividness of their visual imagery on a five-point rating scale, with the following response options: “1 - No image at all, you only ‘know’ that you are thinking of an object,” “2 - Vague and dim,” “3 - Moderately clear and vivid,” “4 - Clear and reasonably vivid,” and “5 - Perfectly clear and vivid as normal vision.” The VVIQ has therefore proved useful in distinguishing individual differences in visual imagery abilities, and these can be categorized along a visual imagery ability spectrum (Zeman et al., 2020).

At one end of the visual imagery ability spectrum, Zeman et al. (2015) coined the term *aphantasia* to describe individuals who self-report an inability or difficulty in generating visual imagery. More recently, it has been proposed that the use of the term aphantasia should be restricted to describe only individuals who self-report a complete inability to generate visual imagery (Blomkvist and Marks, 2023; Reeder and Figueroa, 2022). *Hypophantasia* has been proposed to describe individuals who report the ability to only generate vague and dim visual imagery (Reeder and Figueroa, 2022). At the other end of the spectrum, the term *hyperphantasia* has been used to describe individuals who self-report the ability to generate visual images that are as vivid as real vision (Zeman et al., 2020). However, most of the general population fall between the extreme ends of the spectrum and can be described as having “typical” imagery ability (i.e., phantasia), in that they self-report the ability to generate moderately clear or clear visual imagery (Zeman et al., 2015).

Emerging evidence indicates that differences in visual imagery ability may be underpinned by neurophysiological differences and may contribute to various psychological and behavioral functions (see Pearson, 2019). For example, functional magnetic resonance imaging (fMRI) research by Milton et al. (2021) revealed stronger connectivity between prefrontal brain regions and the visual network during resting state. Similarly, during task-based fMRI greater activity was reported in individuals with hyperphantasia than with aphantasia, specifically in the left anterior parietal region during visual imagery of famous faces and places. Corticospinal excitability, assessed through transcranial magnetic stimulation, is also increased during motor imagery in participants with typical imagery ability, but not in those who experience aphantasia (Dupont et al., 2023). Furthermore, when required to imagine bright and dark visual stimuli individuals with aphantasia do not exhibit pupil constriction and dilation, respectively, as occurs in individuals with typical imagery ability (Kay et al., 2022).

At a cognitive level, reduced visual imagery generation ability may be linked to certain memory deficits. For example, participants with aphantasia recalled fewer internal details during an autobiographical memory interview than those with hyperphantasia (Milton et al., 2021). People with aphantasia also appear to use different, less visual, strategies to those with typical imagery abilities on visual working memory tasks, despite achieving comparable performance (Keogh et al., 2021). Reduced visual imagery abilities (i.e., aphantasia and hypophantasia) have also been associated with positive psychological effects, such as fewer intrusive memories following exposure to trauma (Keogh et al., 2023), indicating a possible protective factor of lower visual imagery abilities against post-traumatic stress disorder. There is also some evidence that visual imagery abilities may be associated with specific career pathways, with reports that aphantasia may be associated with increased propensity to pursue scientific or mathematical professions, while hyperphantasia may be linked to more artistic professions (Zeman et al., 2020).

As visual imagery plays a role in many cognitive and behavioral tasks fundamental to daily living, it is somewhat surprising that the relative prevalence of differing visual imagery abilities and the criteria for distinguishing them remains poorly established. For example, Betts (1909) administered an imagery ability questionnaire to 143 participants across four studies. Although the seven-point rating scale used makes it difficult to compare directly with the five-point rating scale of the VVIQ, based on the scale labels used in the questionnaire, averages across Betts’ four studies indicate a prevalence of 8.25% for what would now be recognized as aphantasia, 24% for hypophantasia, 52% for typical imagery ability, and 15.75% for hyperphantasia. A century later, Faw (2009) surveyed 2,500 participants and reported estimates of between 2 and 5% for what could now be recognized as aphantasia/hypophantasia and up to 30% for hyperphantasia. Although informative, these earlier estimates are limited by various methodological issues. For instance, Betts’ (1909) estimates came from a sample of 143 participants (obtained across four separate studies); a sample size unlikely to be adequate for an accurate estimate based on modern sample size calculators for prevalence studies (e.g., Naing et al., 2022). These estimates also ranged considerably across the four studies (e.g., 2–19% for aphantasia), which may be indicative of some inconsistency or bias in recruitment, sampling, or testing procedures (Dance et al., 2022). In addition, Faw’s (2009) estimates are based on participant responses to a single item that asked participants to rate the general clarity they experience when trying to form a mental image. This may have produced inflated estimates, as recent evidence indicates that single item ratings of imagery ability produce considerably higher prevalence estimates compared to when responses are obtained via validated multi-item questionnaires (Beran et al., 2023).

In recent studies, some of the limitations associated with past prevalence estimates have been overcome as newer estimates are based on responses to the multi-item VVIQ. In a study of VVIQ responses from 1,288 UK-based participants, prevalence estimates of 0.7% for extreme aphantasia (based on a minimum VVIQ score of 16), 2.6% for aphantasia (VVIQ scores of 16–23), 11.2% for hyperphantasia (VVIQ scores of 75–80), and 2.6% for extreme hyperphantasia (based on a maximum VVIQ score of 80) were reported (Zeman et al., 2020). Similarly, Dance et al. (2022) collected VVIQ responses from 1,004 participants based in the UK and United States and calculated a prevalence of 3.9% for aphantasia (comprising 0.8% with a VVIQ score of 16 and 3.1% within a VVIQ range of 17–32). However, estimates for hyperphantasia were not reported. Most recently, in a study with a sample of 5,010 participants from the United States, an aphantasia prevalence estimate of 8.9% was reported when individuals responded to a single-item rating, yet this estimate dropped to 1.5% based on VVIQ scores of 16–23 (Beran et al., 2023). Although not reported explicitly by Beran et al. (2023), their data also suggests a hyperphantasia prevalence estimate of 5.9% (based on 298 out of 5,010 participants scoring within a 75–80 VVIQ range).

Despite some consistency in measurement scales (i.e., the VVIQ) in recent work, comparison between studies is still difficult given the use of different, and often seemingly arbitrary, VVIQ score ranges to determine the imagery ability categories (Blomkvist and Marks, 2023). For example, both Zeman et al. (2020) and Dance et al. (2022) provided prevalence estimates for aphantasia of 0.7 and 0.8%, respectively, based on a minimum VVIQ score of 16, indicative of a total absence of visual imagery ability. However, no such estimate for a complete absence of visual imagery ability was provided by Beran et al. (2023). Furthermore, the studies used different VVIQ score ranges to define a broader aphantasia category. Zeman et al. (2020) and Beran et al. (2023) categorized participants as experiencing aphantasia if they scored between 16 and 23. This range therefore included participants who self-reported experiencing no visual imagery ability whatsoever (i.e., scores of 16) and participants who self-reported experiencing vague and dim visual imagery in response to some VVIQ items (i.e., scores of 17–23). The reported estimates using this range, 2.6 and 1.5% respectively, are therefore based on a combination of participants who could be considered to experience aphantasia or hypophantasia, but crucially do not provide an accurate representation of the true prevalence of those who consistently experience vague and dim visual imagery (i.e., hypophantasia) in response to all VVIQ items (scores ranging from 17 to 32). Where this broader 17–32 range has been applied, an estimate of 3.1%, for what could be considered hypophantasia, has been reported (Dance et al., 2022).

The use of arbitrary cut-offs to assess the prevalence of visual imagery ability categories is clearly problematic (Blomkvist and Marks, 2023) and the use of consistent criteria across studies would be beneficial. At the lower end of the spectrum, a VVIQ score of 16 would, qualitatively, be interpreted as experiencing no voluntary visual imagery for all VVIQ items. This seems an appropriate criterion to categorize aphantasia as the ‘a’ prefix denotes an absence of visual imagery ability (Zeman et al., 2015). Similarly, if hypophantasia is defined as the ability to generate vague and dim visual imagery (Reeder and Figueroa, 2022), VVIQ scores ranging from 17 to 32, as used by Dance et al. (2022), would be appropriate, as this range would comprise responses of no imagery and vague and dim visual imagery to all VVIQ items. As hyperphantasia is used to describe visual imagery that is as vivid as normal vision (Zeman et al., 2020), it seems reasonable to categorize this using VVIQ scores in the range of 75–80, as used by Zeman et al. (2020), as this would capture individuals reporting visual imagery as vivid as normal vision to the majority of VVIQ items.

In addition to issues regarding how the visual imagery ability categories should be defined, the previous prevalence estimates are limited by several other factors. There has been a primary focus on the lower end of the visual imagery ability spectrum, with prevalence data lacking (or at least not explicitly reported) concerning typical imagery ability or hyperphantasia (Beran et al., 2023; Dance et al., 2022). Although the prevalence of hyperphantasia has been detailed by Zeman et al. (2020), this study may be subject to sampling bias due to the mentioning of visual imagery explicitly in the study recruitment materials (Dance et al., 2022). A reliable estimate of the prevalence of hyperphantasia, therefore, remains to be established. In addition, none of the more recent studies (Zeman et al., 2020; Dance et al., 2022; Beran et al., 2023) have reported a sample size calculation to confirm an adequately sized sample for a prevalence estimate. This is problematic considering that the only studies to report prevalence estimates for aphantasia based on a minimum VVIQ score of 16 (Zeman et al., 2020; Dance et al., 2022) or hypophantasia based on a VVIQ range of 17–32 (Dance et al., 2022) may not have an appropriately sized sample for an accurate prevalence estimate (see “Participants and sample size calculation” section below). Researchers to date have also only recruited participants from relatively narrow geographic locations, sampling from only one or two countries. As such, international prevalence estimates for differing visual imagery ability categories remain to be established.

The aim of the current research was to establish prevalence estimates for different visual imagery abilities across different geographical locations. This aim was achieved across two studies. In Study 1, the VVIQ was administered to an appropriately sized sample for prevalence estimates, covering a wider range of geographical locations than has been studied previously (i.e., in addition to the UK and United States). A secondary aim of Study 1 was to establish how various demographic factors such as age, gender, education, and nationality, may be associated with differing visual imagery abilities. To further establish prevalence, in Study 2, data was collated from previous studies investigating the aphantasia prevalence and combined with the participant sample from Study 1. From this larger sample, prevalence estimates are provided for aphantasia, hypophantasia, typical imagery ability, and hyperphantasia.

## Study 1

### Method

#### Participants and sample size calculation

**Figure 1:**
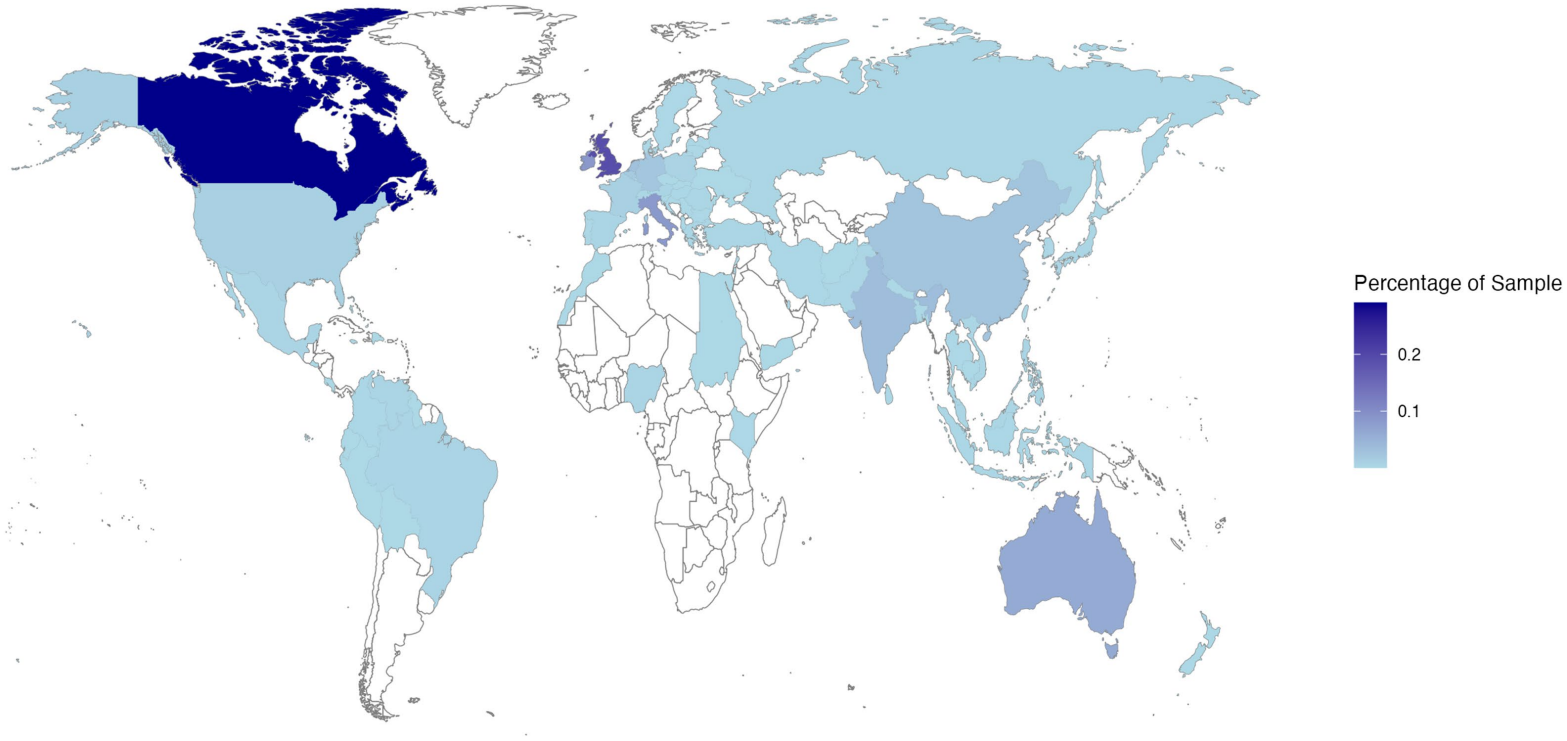
Heatmap showing the distribution of Study 1 participant reported nationalities on a world map (via R Programming Environment using https://CRAN.R-project.org/package=maps). Darker shades of blue indicate that a greater proportion of the sample represented a particular nationality.

The final sample comprised 3,049 participants. The mean age was 27.04 years (SD = 11.74 years), with a gender split of 2,197 females, 758 males, 57 non-binary, 9 gender fluid, 9 transgender, 2 of another gender identity, and 17 individuals who preferred not to state their gender identity. Participants represented 85 nationalities (see Figure 1) and self-reported being qualified or currently studying at the following educational levels: doctoral = 5.2%, postgraduate = 18.4%, undergraduate = 68.3%, further education = 6.0%, secondary education = 1.3%, preferred not to say = 0.8%.

The required sample size was determined using the Scalex SP sample size calculator for prevalence studies (Naing et al., 2022). Based on an expected aphantasia prevalence of approximately 4% (Dance et al., 2022), a sample size of 1969 was required to achieve the desired precision of ±1% in estimating the prevalence with 95% confidence, while accounting for potential data loss of 25%. The final sample size exceeded this target considerably due to simultaneous multi-site recruitment, ensuring an adequate sample size for the prevalence estimates.

### Materials

#### Demographic questions

Participants self-reported demographic information regarding their age, gender identity, educational level, nationality, and language fluency.

#### Vividness of visual imagery ability

Visual imagery ability was measured using the 16-item Vividness of Visual Imagery Questionnaire (VVIQ; Marks, 1973), a valid and reliable measure of conscious ability to generate vivid visual imagery (Marks, 1999; Campos and Pérez-Fabello, 2009). This questionnaire directs participants to generate a visual image of four different scenarios and rate the vividness of their visual imagery concerning four aspects of each scenario. For example, one scenario instructs participants to “Think of some relative or friend whom you frequently see (but who is not with you at present) and consider carefully the picture that comes before your mind’s eye.” Participants are then instructed to rate the vividness of their visual imagery regarding “The exact contour of face, head, shoulders and body” and “The different colors worn in some familiar clothes.” Participants provide their visual imagery vividness ratings on a five-point rating scale, with the following response options: 1 = “No image at all, you only ‘know’ that you are thinking of an object,” “2 = Vague and dim,” “3 = Moderately clear and vivid, “4 = Clear and reasonably vivid,” “5 = Perfectly clear and vivid as normal vision.” Note that as is common in modern studies using the VVIQ (e.g., Dance et al., 2022; Milton et al., 2021), this scoring system was reversed from the original to more intuitively reflect lower scores indicating lower visual imagery ability and higher scores indicating higher visual imagery ability. To score the questionnaire, responses to each item were totaled to produce scores ranging from 16 (responses of 1 to all items) to 80 (responses of 5 to all items). Four additional attention check items were embedded to create a 20-item survey in total. One attention check was included after each of the four items related to a particular imagery scenario. The attention checks directed participants to confirm their attention by selecting a specific item on the response scale (e.g., “To confirm that you are paying attention, please select ‘3 – Moderately clear and vivid’”) and were used to identify participants responding without due care and attention for removal from the sample.

### Procedure

Ethical approval for the study was obtained from the Health and Education Research Ethics and Governance Committee at Manchester Metropolitan University (Ethical approval number: 42688). Identical English, French, German, and Italian language versions of the study materials were created in Qualtrics (Qualtrics, Provo, UT). As well as the original English language version of the VVIQ (Marks, 1973), published translations of the VVIQ were used for French (Santarpia et al., 2008), German (Jungmann et al., 2022), and Italian (Talamini et al., 2022) languages. All other recruitment media and study materials were translated from English by native multilingual speakers on the research team (BW - French; SD - German; MB - Italian) and, in addition, reviewed for accuracy by at least one other native speaker. Responses to demographic questions indicated that all participants self-reported fluency in the language in which they completed the survey.

Members of the research team distributed the link or QR code to the Qualtrics survey as widely as possible via (i) in-class or email announcements to student cohorts, (ii) inclusion on university research participation pools, (iii) social media advertisements, (iv) personal contacts, and (v) word-of-mouth. In line with the procedures of Dance et al. (2022), and to ensure a reliable prevalence estimate, recruitment announcements contained no mention of terms related to visual imagery ability (e.g., visual imagery, aphantasia, hyperphantasia), and instead invited participants to take part in a “psychology project exploring cognitive processes.” This was done to (i) avoid biasing the sample by encouraging/discouraging participation from individuals who were already aware of their extreme imagery abilities, and (ii) reduce the possibility of demand characteristics influencing participants’ responses.

Upon accessing the survey link, participants were presented with an information sheet outlining what participation would involve, again worded as a project exploring cognitive processes and without using terms related to visual imagery. Participants then provided informed consent to take part and were instructed to create a unique participant identification code, against which their data was recorded anonymously. Following this, participants responded to the demographic questions before completing the VVIQ. A debrief page was presented upon completion of the survey. The debrief outlined the full aim of the study (i.e., to establish prevalence estimates for aphantasia and hyperphantasia), provided a link to the Aphantasia Network^1^ for participants who may have wanted more information, and provided instructions for how participants could withdraw their data from the study if they wished. The median completion time for the survey was 6 min 28 s.

### Data processing and analysis

In total the link to the survey was opened 4,544 times. Filtering out non-completions resulted in the removal of 1,169 responses, while identification of duplicate responses resulted in a further 235 responses being removed. Participants who failed any of the attention check questions were then identified, which resulted in the removal of a further 91 responses and a final sample of 3,049 participants.

The data were analyzed with IBM SPSS Statistics (Version 28). Cronbach’s alpha was calculated to determine the internal consistency of the VVIQ. Participants were then classified as experiencing aphantasia if they reported a score of 16 on the VVIQ, while participants who scored between 17 and 32 on the VVIQ were classified as experiencing hypophantasia. Those who reported scores between 33 and 74 were classified as having typical visual imagery ability, while those who scored 75–80 were classified as experiencing hyperphantasia. Descriptive statistics on the frequencies of each visual imagery ability classification were expressed as percentages and 95% confidence intervals were calculated.

Further exploratory analyses were performed to identify whether the visual imagery ability was influenced by the demographic variables of age, gender, education, and nationality. Those who preferred not to state their age (*n* = 4), gender identity (*n* = 17), education level (*n* = 24), or nationality (*n* = 5) were removed from these respective analyses. A Pearson’s correlation was conducted to establish whether total VVIQ scores correlated significantly with age. For this correlation, aphantasics scoring 16 on the VVIQ, indicative of no visual imagery ability, were removed. This ensured that the correlation reflected how the ability to imagine visually varied across age and the magnitude of this potential relationship was not diminished by the inclusion of those without the ability to generate voluntary visual imagery. Separate Chi-square analyses were conducted to establish whether the prevalence of each visual imagery category varied as a function of gender identity, education level, or nationality. For the Chi-square tests, examination of the outputs from the initial analyses revealed that the assumption of expected cell count was violated due to the numbers in certain groups returning expected counts below five (McHugh, 2013). To avoid violating this test assumption, for the gender analysis, only male and female gender identities were included. Similarly, for the education analysis, education levels were re-grouped into the following four categories: doctoral degree level, postgraduate degree level, undergraduate degree level, pre-university level. Finally, for the nationality analysis, nationalities were re-grouped based on continents into the following categories: Asian, North American, European, Australasian (see Table 1 for a breakdown of visual imagery categories by nationalities with >100 participants).

**Table 1:** Breakdown of visual imagery categories by participant nationality for Study 1 (for nationalities where there were >100 participants per nationality).

Nationality		Aphantasic	Hypophantasic	Typical imagery	Hyperphantasic	Total
Australian	Total	6	7	160	10	183
	%	3.28	3.83	87.43	5.46	100
British	Total	11	26	521	23	581
	%	1.89	4.48	89.67	3.96	100
Canadian	Total	15	32	790	50	887
	%	1.69	3.61	89.06	5.64	100
Indian	Total	1	1	95	5	102
	%	0.98	0.98	93.14	4.90	100
Irish	Total	3	5	229	21	258
	%	1.16	1.94	88.76	8.14	100
Italian	Total	1	1	239	12	253
	%	0.40	0.40	94.47	4.74	100
Maltese	Total	1	4	91	12	108
	%	0.93	3.70	84.26	11.11	100

### Results

#### Internal consistency analysis

A Cronbach’s alpha calculation revealed *α* = 0.926, indicating excellent internal consistency for the VVIQ (Gliem and Gliem, 2003).

#### Prevalence estimates

**Figure 2:**
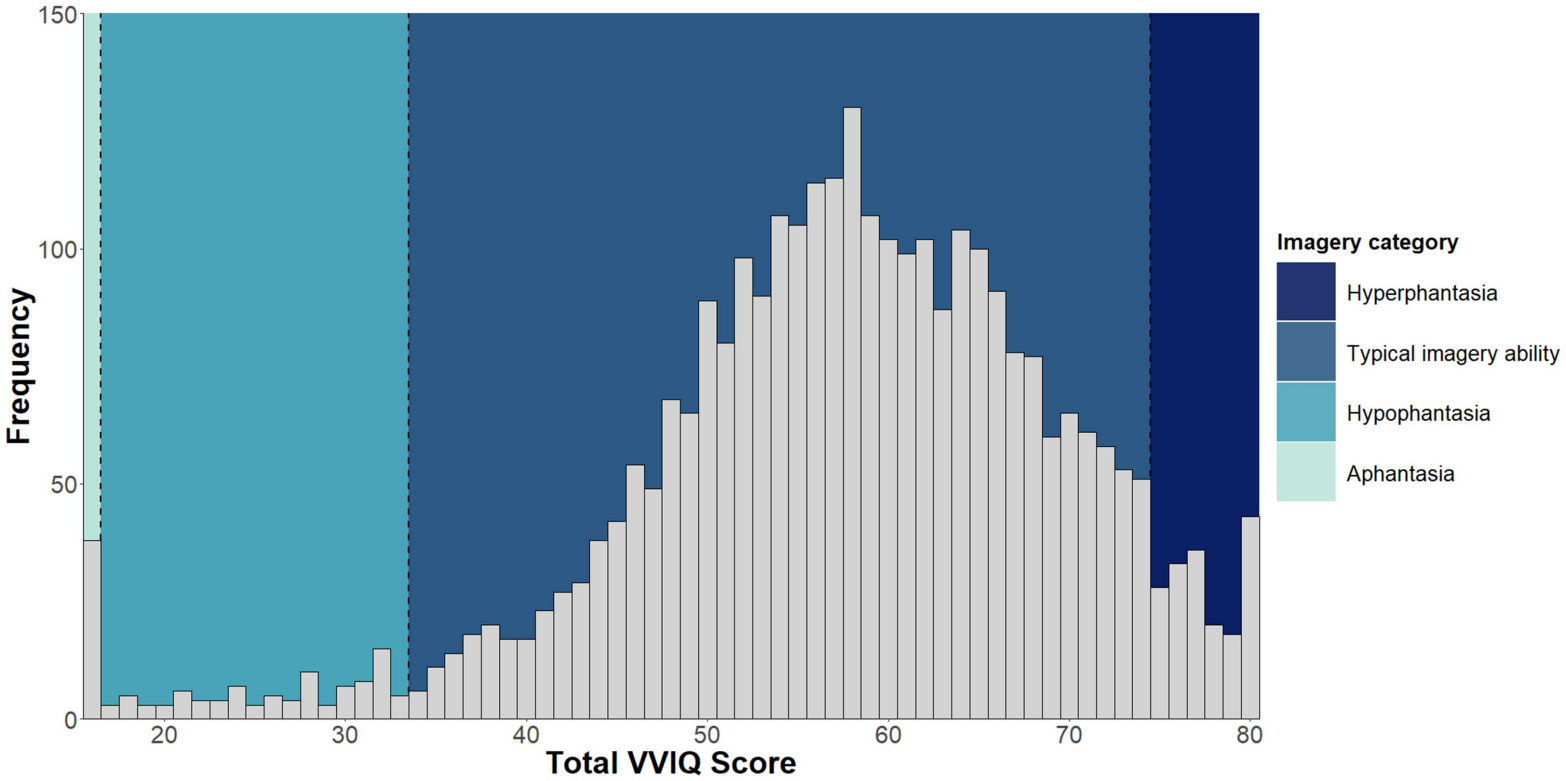
Histogram showing the distribution of VVIQ scores across the Study 1 sample. Visual imagery ability categories (Aphantasia, Hypophantasia, Typical imagery ability, and Hyperphantasia) are color coded to illustrate the frequency of responses for each category.

The median VVIQ score for the sample was 58 (IQR = 51–66). The distribution of VVIQ scores across the sample is shown in Figure 2. Within the sample of 3,049 participants, 38 were classified as experiencing aphantasia (*M* age = 27.45, ± 12.42; 29 female, 8 male, 1 non-binary). This corresponds to a 1.2% (95% CI [0.9, 1.7]) prevalence estimate for aphantasia. A further 90 participants (*M* age = 26.71, ±11.03; 65 female, 18 male, 4 non-binary, 1 gender fluid, 1 transgender, 1 other gender) were classified as experiencing hypophantasia, equating to a 3.0% (95% CI [2.4, 3.6]) prevalence estimate. If combined, a prevalence estimate of 4.2% can be calculated for individuals who self-report either a complete absence of visual imagery ability or difficulty generating visual imagery. Most participants (*N* = 2,742; *M* age = 26.91, ±11.61; 1975 female, 686 male, 50 non-binary, 7 gender fluid, 8 transgender, 16 who preferred not to state their gender identity) were classified as having typical imagery ability, corresponding to a prevalence estimate of 89.9% (95% CI [88.8, 91.0]). The remaining 179 participants (*M* age = 29.04, ±13.75; 128 female, 46 male, 2 non-binary, 1 gender fluid, 1 other gender, 1 who preferred not to state their gender identity) were classified as experiencing hyperphantasia, which equates to a prevalence estimate of 5.9% (95% CI [5.1, 6.8]) for hyperphantasia. This hyperphantasia estimate comprises 4.5% (95% CI [3.8, 5.3]) with VVIQ scores between 75 and 79, and 1.4% (95% CI [1.0, 1.9]) with a maximum VVIQ score of 80.

#### Exploratory analyses

**Figure 3:**
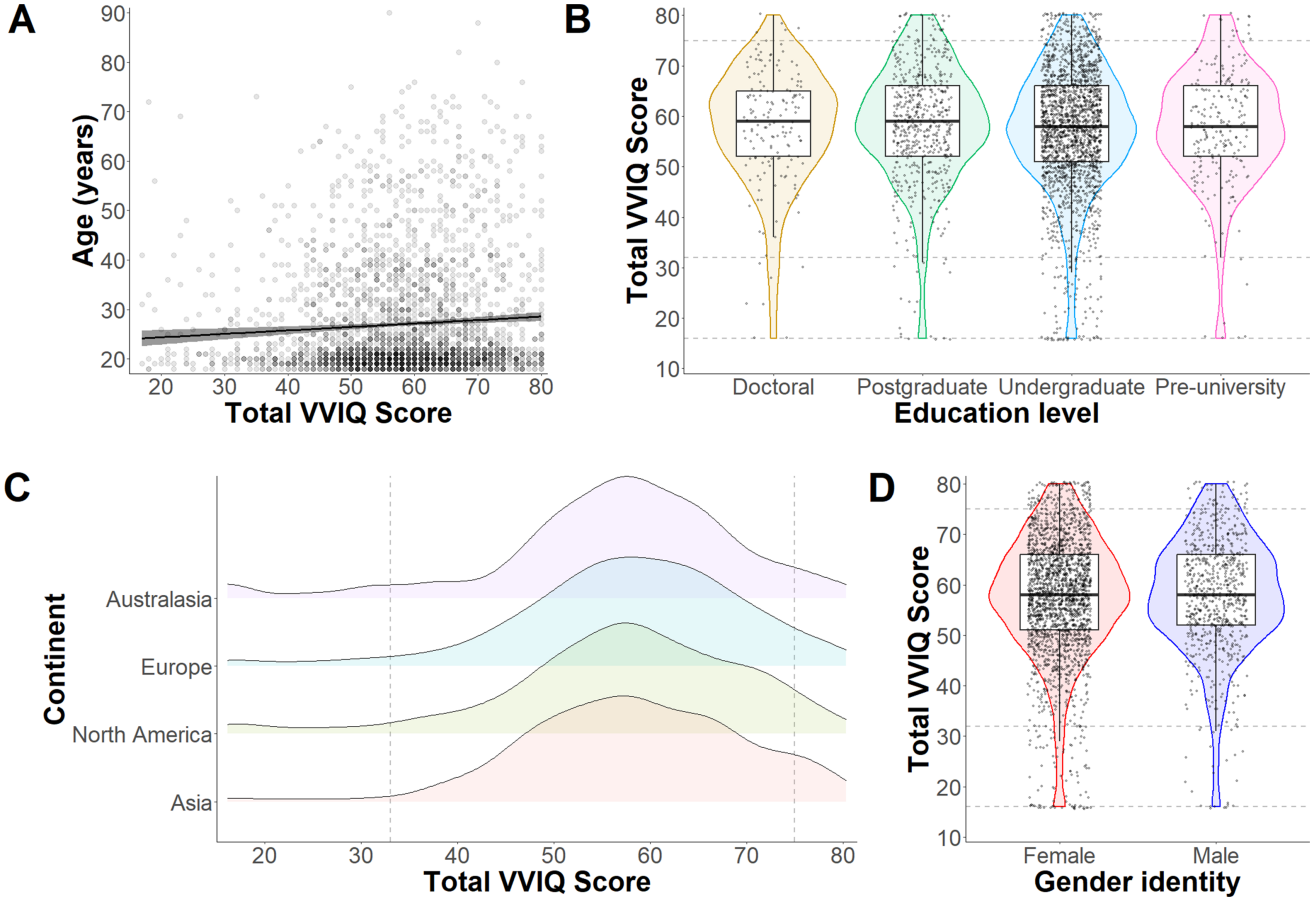
Exploratory analyses from Study 1. Panel **(A)** illustrates the correlation between total VVIQ scores (without Aphantasia scores) and age (years) across all participants. The gray band around the regression line represents 95% confidence intervals. The violin plots in Panel B depict distributions, medians and quantiles for VVIQ responses depending on level of education (Doctoral, Postgraduate, Undergraduate or Pre-university). Individual points represent individual participant responses and dashed lines intercepting on the y axis represent boundaries for Aphantasia, Hypophantasia, Typical imagery ability, and Hyperphantasia (bottom to top). The density plots in Panel **(C)** represent response distributions for each continent (Australasia, Europe, North America, and Asia). Here the figure starts at 16 (representing Aphantasia) and the dashed lines intercepting on the x axis represent the other imagery ability boundaries (as in Panel **B**). Finally, Panel **(D)** shows distributions, medians and quantiles of responses based on gender identity (yet restricted to only female and male to avoid violating chi square test assumptions).

There is currently limited information available on how visual imagery abilities vary as a function of demographic characteristics. As such, exploratory analyses were conducted to examine the influence of age, education level, nationality, and gender on visual imagery ability. As illustrated in Figure 3A, there was a weak but significant positive correlation between age and total VVIQ score, *r* (3043) = 0.06, *p* = 0.001. Chi-square tests revealed no significant associations between education level and visual imagery ability category, χ^2^ (9, *N* = 3,025) = 4.96, *p* = 0.838, between continent-based nationality and visual imagery ability category, χ^2^ (9, *N* = 2,975) = 15.020, *p* = 0.090, or between gender identity and visual imagery ability category, χ^2^ (3, *N* = 2,955) = 1.078, *p* = 0.782 (see Figures 3B–D).

## Study 2

The findings of Study 1 provide prevalence estimates for visual imagery ability categories from an international sample. The aim of Study 2 was to enhance the accuracy of these estimates by replicating the analyses on a larger sample. This was achieved by combining the data from Study 1 with data available openly from previous aphantasia prevalence studies (Beran et al., 2023; Dance et al., 2022) and re-calculating the prevalence estimates.

### Method

The VVIQ data collected in the aphantasia prevalence studies by Dance et al. (2022) and Beran et al. (2023) are available openly on the Open Science Framework. The dataset from the study by Dance et al.^2^ contains VVIQ scores from 1,004 participants who were based in the UK or United States, and the dataset from the study by Beran et al.^3^ contains VVIQ data from 5,010 participants from the United States. Combining these data with data from Study 1 produced a total sample of 9,063 participants. A chi-square analysis was conducted to establish whether the proportion of participants in each visual imagery category varied across the three studies from which data were taken (Beran et al., 2023; Dance et al., 2022; Study 1). Revised prevalence estimates were calculated for aphantasia (VVIQ score = 16), hypophantasia (VVIQ score = 17–32), typical imagery ability (VVIQ score = 33–74), and hyperphantasia (VVIQ score 75–80) using this larger sample.

### Results

**Figure 4:**
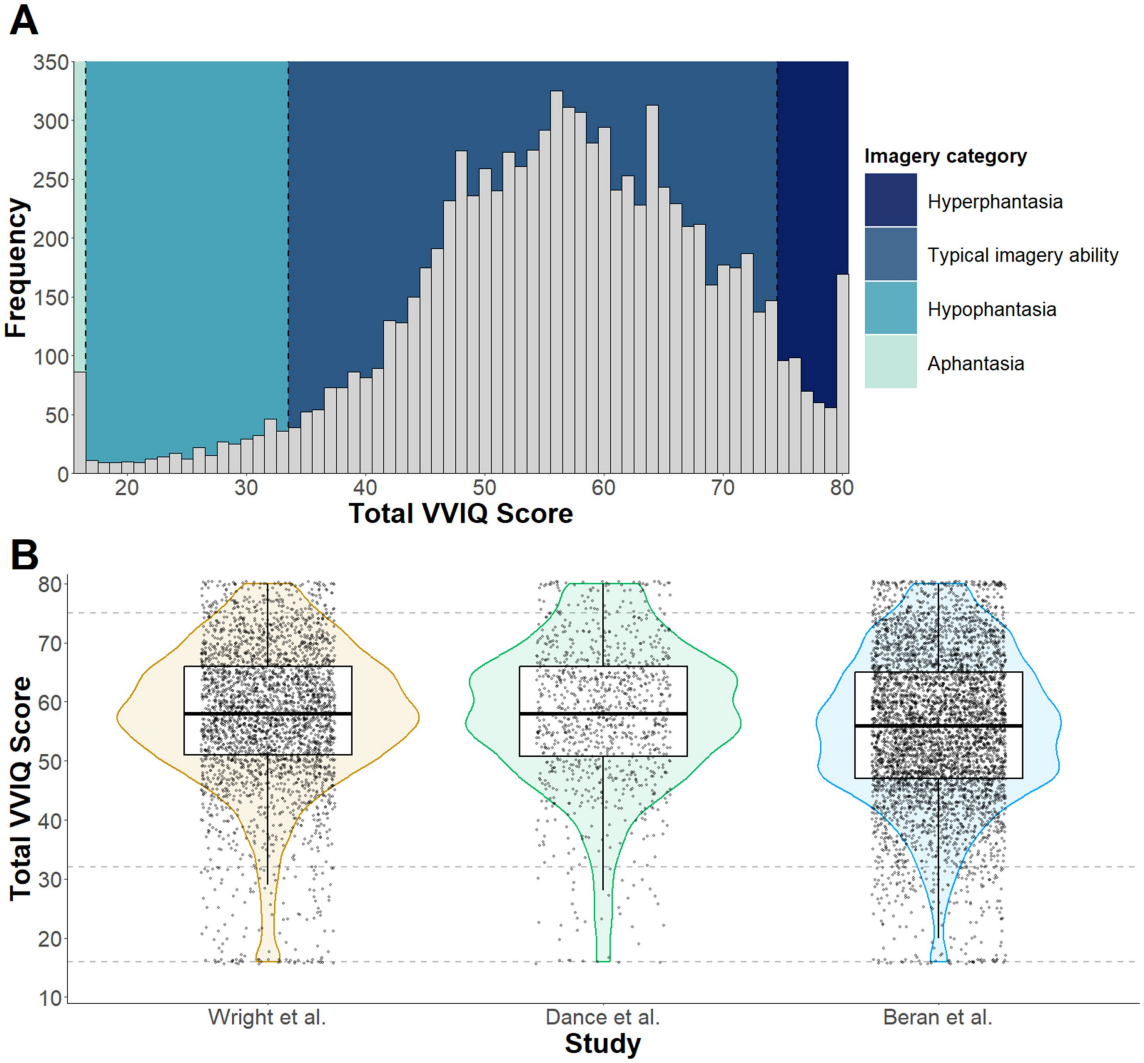
Panel **(A)** depicts a histogram of VVIQ scores across the combined samples collected by Dance et al. (2022), Beran et al. (2023) and Study 1 of the current paper. Visual imagery ability categories (Aphantasia, Hypophantasia, Typical imagery ability, and Hyperphantasia) are color coded to illustrate the frequency of responses within each category. The violin plots in Panel **(B)** depict sample distribution, median and quantile for each dataset included in Study 2, with the dashed lines intercepting on the y axis representing boundaries for imagery categories.

The chi-square test indicated that the proportion of participants in each visual imagery ability category did not vary across Study 1 and the Beran et al. (2023) and Dance et al. (2022) studies, χ^2^ (6, *N* = 9063) = 12.21, *p* = .058. The median VVIQ score for the combined sample was 57 (IQR = 49–65). Re-calculation of the prevalence estimates produced similar results to those reported in Study 1 (see Figure 4). Within the sample of 9,063 participants, 86 were classified as experiencing aphantasia, equating to a 0.9% (95% CI [0.8, 1.2]) prevalence estimate. A further 299 participants scored within the hypophantasia category, equating to a 3.3% prevalence estimate (95% CI [3.0, 3.7]). If combined, a prevalence estimate of 4.2% can be calculated for individuals who self-report either a complete absence of visual imagery ability or difficulty generating visual imagery. Scores corresponding to typical imagery ability were reported by 8,129 participants, which equates to 89.7% (95% CI [89.1, 90.3]) of the sample. There were also 549 participants who reported scores within the hyperphantasia range, which equates to a 6.1% (95% CI [5.6, 6.6]) hyperphantasia prevalence estimate. This hyperphantasia estimate comprises 4.2% (95% CI [3.8, 4.6]) with VVIQ scores between 75 and 79, and 1.9% (95% CI [1.6, 2.2]) with a maximum VVIQ score of 80.

## Discussion

The primary aim of this research was to establish international prevalence estimates based on responses to the VVIQ for the following visual imagery ability categories: aphantasia (VVIQ score = 16), hypophantasia (VVIQ score = 17–32), typical imagery ability (VVIQ score = 33–74), hyperphantasia (VVIQ score = 75–80). Study 1 revealed prevalence estimates of 1.2% for aphantasia, 3.0% for hypophantasia, 89.9% for typical imagery ability, and 5.9% for hyperphantasia, based on an international sample of 3,049 participants. Study 2 involved combining the Study 1 data with openly available data from studies by Dance et al. (2022) and Beran et al. (2023) and applying the same analysis on a larger sample of 9,063 participants. This returned similar prevalence estimates to Study 1 of 0.9% for aphantasia, 3.3% for hypophantasia, 89.7% for typical imagery ability, and 6.1% for hyperphantasia. This is the largest-scale prevalence estimate for different visual imagery ability categories conducted to date and the first to be informed by a sample size calculator for prevalence studies (Naing et al., 2022). The findings reported here provide the most robust prevalence estimates of differing visual imagery ability categories available to date and provide a platform for future studies investigating various cognitive, behavioral, and neuroscientific tasks that involve visual imagery.

At the lower end of the visual imagery ability spectrum, early estimates provided by Betts (1909) for what would now be described as aphantasia (8.25%) and hypophantasia (24%) are considerably higher than prevalence estimates reported in this study. However, these earlier estimates were based on a sample of 143 participants; less than 10% of the sample size recommended based on Naing et al.’s (2022) sample size calculator for prevalence studies. Betts’ estimates were also based on averages that varied widely across four separate studies, with some likely over-inflated due to recruitment bias or other methodological issues (Dance et al., 2022). Faw’s (2009) estimates for aphantasia (2–5%) and hypophantasia (8.2%) are again higher than the estimates reported here, but this may reflect the use of a single item for the prevalence estimate, rather than use of a multi-item questionnaire like the VVIQ (Dance et al., 2022; Beran et al., 2023). The prevalence estimates reported in more recent studies by Dance et al. (2022) and Zeman et al. (2020) using a VVIQ score of 16 to categorize aphantasia are remarkably similar to those reported here. For example, aphantasia estimates of 0.7% (Zeman et al., 2020) and 0.8% (Dance et al., 2022) correspond closely to the 1.2% estimate reported in Study 1 and the 0.9% estimate reported in Study 2. Estimates for the prevalence of individuals who experience vague and dim visual imagery (i.e., hypophantasia) were less well established in the literature. Beran et al.’s (2023) and Zeman et al.’s (2020) estimates of 1.5 and 2.6% respectively, using a VVIQ range of 16–23, are imprecise due to their inclusion of participants who self-reported aphantasia (VVIQ score of 16) and their exclusion of participants with VVIQ scores of 24–32, reflective of vague and dim visual imagery for all VVIQ items. When applying a VVIQ range of 17–32 to categorize hypophantasia, the estimates from Study 1 (3.0%) and Study 2 (3.3%) correspond closely to the 3.1% estimate reported by Dance et al. (2022). Taken together, there is very strong evidence that approximately 1% of individuals self-report experiencing a complete absence of visual imagery ability, while a further 3% are only able to generate vague and dim visual images. Collectively, this indicates that 4% of the population experience difficulties in generating visual imagery. This finding has implications for both research and applied settings, where experimental tasks or therapeutic interventions involving imagery may be inaccessible for the approximately one in 25 of the general population who experience reduced visual imagery ability; underlining the importance of measuring participant imagery ability characteristics in such settings prior to administering the imagery task (see Moreno-Verdú et al., 2024).

Prevalence estimates at the other end of the visual imagery ability spectrum are less well documented in the literature. Estimates for what would now be considered hyperphantasia of 15.75% (Betts, 1909) and 30% (Faw, 2009) have been reported; considerably higher than that reported in Study 1 (5.9%) and Study 2 (6.1%). These earlier estimates, however, are limited by the study design issues discussed previously. In more recent studies, Dance et al. (2022) and Beran et al. (2023) focused primarily on the lower end of the visual imagery ability spectrum and so did not provide exact prevalence estimates for hyperphantasia. Zeman et al. (2020), however, reported a prevalence estimate of 11.2% for hyperphantasia, based on the same 75–80 VVIQ score range used in the current study. However, the lower estimate of approximately 6% reported here is likely more accurate given the involvement of an adequate sample size and/or the deliberate avoidance of terminology related to visual imagery in the recruitment materials. Based on this large sample, it can be claimed with confidence that around 6% of individuals self-report the ability to generate visual imagery that is as clear and vivid as real vision.

To date, there has been relatively little exploration of different factors that may influence visual imagery ability. The exploratory analyses conducted in Study 1 addressed this gap by examining the relationships between visual imagery ability and demographic variables of age, gender, education level, and nationality. The findings indicated a weak but significant increase in visual imagery ability with increasing age. This supports evidence that older individuals are more likely than younger individuals to report VVIQ scores within the hyperphantasia range (Beran et al., 2023), yet elsewhere a decline in visual imagery ability has also been associated with increased age (Gulyás et al., 2022). Given the inconsistent findings, further research exploring how visual imagery ability develops with age and potentially changes over the lifespan would be worthwhile. This research would help to provide insights into the stability of visual imagery ability as well as reasons as to why it might change, based on other cognitive or sensory-motor metrics that experience co-comitant age-related changes.

The gender analysis showed no significant association between gender and visual imagery ability category. This null finding aligns with previous prevalence studies that have also not identified differences in the prevalence of aphantasia across sex or gender (Beran et al., 2023; Dance et al., 2022). Despite some evidence that females may experience more vivid visual imagery than males (e.g., Isaac and Marks, 1994), the current body of data does not support the existence of gender-based differences in the prevalence of aphantasia, hypophantasia, or hyperphantasia, at least between traditional binary gender identities that have been examined statistically to date. Neither does it seem that visual imagery ability impacts (or is impacted by) education level. There is some evidence that individuals who experience aphantasia and hypophantasia perform less well on memory tests than those with typical imagery ability or hyperphantasia (Beran et al., 2023; Milton et al., 2021). However, such visual imagery deficits and associated interference on memory tests do not seem to have further reaching implications on educational trajectories or grades (Monzel et al., 2023). Our data tentatively supports this conclusion with no association between imagery ability and education level, and thus may reflect those with lower visual imagery abilities adapting to use different and less visual memory strategies to achieve comparable performance (Keogh et al., 2021). Future researchers, however, may wish to further explore whether visual imagery abilities influence other educational variables beyond level of study or grades, such as literacy or numeracy skills.

Regarding nationality, previous studies have recruited samples from relatively narrow geographic locations based on one or two countries. Beran et al. (2023) acknowledged this limitation and called for future research to explore visual imagery ability categories across a wider range of populations. Despite this wider capture in Study 1, there was no evidence of continent-based nationality differences in the relative prevalence of different visual imagery ability categories across Asia, North America, Europe, and Australasia. Participants who experienced aphantasia, hypophantasia, and hyperphantasia were found across these geographic locations (see Table 1 for a breakdown of visual imagery categories by nationalities with > 100 participants), providing the first evidence that these individual differences in visual imagery abilities are prevalent across different international locations and cultures. It should be noted, however, that small participant numbers from Africa and South America resulted in their exclusion from this analysis. Further research specifically targeting participants from countries of varying degrees of economic development may be worthwhile to allow firm conclusions to be drawn regarding international variability in visual imagery.

Despite the positive contributions to the literature made by these prevalence estimates, limitations should be acknowledged. As in previous literature, the prevalence estimates were based on responses to a self-report questionnaire. Although the VVIQ measure used in this study is the most common tool for identifying visual imagery ability categories (e.g., Beran et al., 2023; Dance et al., 2022; Zeman et al., 2020), this tool is susceptible to social desirability and demand characteristics. While more objective (e.g., neurophysiological) markers of visual imagery ability exist (e.g., Dupont et al., 2023; Kay et al., 2022; Milton et al., 2021), it would not have been feasible to record these measures from a sample of several thousand participants. However, there is also evidence that VVIQ scores are associated with more objective markers of visual imagery ability (Kay et al., 2022), and so the use of the VVIQ as a self-report measure is appropriate for a large-scale prevalence estimate. Despite this, the VVIQ only measures voluntary visual imagery ability, so does not allow conclusions to be drawn regarding the prevalence of involuntary visual imagery abilities which may also be affected in aphantasia (see Krempel and Monzel, 2024). Furthermore, the findings reported here do not distinguish between cases of aphantasia that are congenital (i.e., from birth) or acquired during the lifespan through injury or cognitive degeneration (Knowles et al., 2021), and so it is not possible to dissociate the prevalence of these two forms of aphantasia in the current study. Lastly, visual imagery abilities may also differ between neurotypical and neurodivergent populations (e.g., Barhoun et al., 2019) but neurodevelopmental status was not accounted for in the results reported here. Given recent research suggesting a link between aphantasia and autism (Dance et al., 2021a), further investigation into whether the prevalence of imagery ability categories differs between the general population and various clinical populations would be worthwhile.

The findings reported here have several important implications for research. There is a growing body of evidence indicating that visual imagery abilities at either end of the visual imagery ability spectrum are associated with various neurophysiological (Dupont et al., 2023; Milton et al., 2021), behavioral (Kay et al., 2022), and psychological (Keogh et al., 2021; Keogh et al., 2023; Milton et al., 2021) differences. Given that these extreme visual imagery abilities make up around 10% of the population (4.2% who experience visual imagery difficulties, and 6.1% who experience visual imagery like real vision), further exploration of the neurophysiological mechanisms underpinning these differing visual imagery abilities, and the psychological effects of these in relation to cognitive processes like memory, creativity, and responses to trauma or bereavement is warranted.

The current findings also have implications for interventions that make use of imagery techniques. For example, visual imagery techniques play an important role in cognitive behavior therapy (Saulsman et al., 2019; Monzel et al., 2024) where it can be used in various ways, including to facilitate emotional coping or assist in the treatment of phobias. In such instances, the 4.2% of the population who struggle to generate visual imagery may conceivably gain little-to-no benefits from such interventions, and so alternative non-imagery-based approaches may be more suitable. For instance, it has recently been found that propositional thought – thinking of stimuli without conjuring an image in the mind’s eye – may be a sufficient replacement for imagery-based components of therapies (Monzel et al., 2024). Similarly, motor imagery interventions involve visual and kinaesthetic imagery of movement (Eaves et al., 2022; Krüger et al., 2024) and are used frequently to support motor skill acquisition in various rehabilitative and sport-based contexts (Frank et al., 2024; Simonsmeier et al., 2020). Again, these interventions may provide little benefit to those who experience aphantasia or hypophantasia. An emerging approach within this field, however, has been to combine action observation with motor imagery (AOMI) by providing visual stimuli on video and instructing participants to simultaneously imagine the feelings of movement execution (Eaves et al., 2016; Scott et al., 2022). This approach reduces the requirement to generate visual imagery and places the emphasis instead on kinaesthetic imagery (Wright et al., 2022), and appears to be at least as effective for motor skill acquisition as traditional motor imagery interventions (Chye et al., 2022). Although there is some evidence that those with reduced visual imagery ability also experience deficits in other imagery modalities (Dance et al., 2021b; Dawes et al., 2020), it is possible that AOMI may offer a more accessible and effective intervention than motor imagery for the 4.2% of the population who experience visual imagery ability impairments. Future research should, therefore, explore the efficacy of motor imagery and AOMI interventions in participants at the lower end of the visual imagery ability spectrum.

In conclusion, this research is the first to provide prevalence estimates for differing visual imagery abilities across the range of the visual imagery ability spectrum, using an appropriately sized sample for a prevalence estimate. Data across two studies show that 4.2% of the population (approximately one in 25 people) struggle to generate visual imagery, with 0.9% unable to do so and 3.3% finding it difficult. At the other end of the visual imagery ability spectrum, 6.1% (approximately one in 16 people) self-report the ability to generate visual imagery that is as clear and vivid as actual vision. These conclusive estimates, based on more than 9,000 adults, should provide clarity within the research literature regarding the prevalence of individuals who experience extreme visual imagery abilities. Future research should now explore the neurophysiology, and antecedents and consequences, of differing visual imagery abilities.

## Data Availability

The raw data supporting the conclusions of this article are available on the Open Science Framework: https://osf.io/t6mg5/?view_only=dc1f1c4e36e542a3b85313072ac90709.

## Footnotes

^1^ https://aphantasia.com

^2^ https://osf.io/mxyv9/?view_only=3f62fbfe59184f6b9c28a3fe4a4375ad

^3^ https://osf.io/ga4ez
